# Serum adiponectin levels are associated with worse cognitive function in postmenopausal women

**DOI:** 10.1371/journal.pone.0186205

**Published:** 2017-12-21

**Authors:** Pasquale De Franciscis, Michelangela Barbieri, Stefania Leo, Anna Maria Dalise, Celestino Sardu, Raffaele Marfella, Nicola Colacurci, Giuseppe Paolisso, Maria Rosaria Rizzo

**Affiliations:** 1 Department of Woman, Child and General and Specialized Surgery, University of Campania “Luigi Vanvitelli”, Naples, Italy; 2 Department of Medical, Surgical, Neurological, Metabolic and Geriatric Sciences, University of Campania “Luigi Vanvitelli”, Naples, Italy; University College London, UNITED KINGDOM

## Abstract

**Introduction:**

Adiponectin may be a biomarker of cognitive impairment stage, and its clinical progression. In this study we aimed to evaluate the relationship between serum adiponectin levels and cognitive performances in menopausal women, and whether serum adiponectin levels may be differently associated with cognitive performances as compared to overweight/obese subjects.

**Methods:**

We enrolled 188 post-menopausal women, divided into two groups: obese/overweight group (n = 96) and normal weight group (n = 92). After a clinical examination, and laboratory measurements, we assessed cognitive functions by Montreal Cognitive Assessment test (MoCA).

**Results:**

A significant and greater decrease in executive/visuo-spatial and in attention functions occurred in obese/overweight group as compared to normal weight group (p< 0.001). A significant positive relationship between serum adiponectin levels, and MoCA Global cognitive function was found. MoCA executive, and MoCA attention functions significantly and positively correlated with serum adiponectin levels. BMI, WHR, and serum adiponectin levels were independently associated with MoCA Global cognitive function, but only serum adiponectin levels were independently associated with MoCA attention.

**Conclusion:**

A significant positive association may exist between serum adiponectin levels, and better cognitive function in postmenopausal status. The major determinant of attentional capacity was just serum adiponectin levels, and dosage of serum adiponectin levels may be early serum marker of cognitive decline. Therefore, serum adiponectin level has to be used, as early biomarker, to detect cognitive decline, and to support an early prevention.

## Introduction

Epidemiological studies suggest a relationship between midlife metabolism and cognitive performances [1–2]. Indeed, in addition to genetic factors, midlife metabolic diseases, as diabetes, hypertension, obesity are independently associated with impaired cognition in old age [3–5]. In female patients, the midlife may be considered as the period of life coinciding with menopause transition. In fact, during menopause transition, many women may experience weight gain, associated to central fat deposition and consequently obesity [6–7]. Recent studies reported that obesity is associated with poor neurocognitive outcome and with structural brain changes, including excess age-related atrophy and white matter disease [8]. Conversely, there is some evidence that obesity may be protective against cognitive decline in women due probably to endogenous estrogens [9–10]. Although the mechanism for gender differences in cognitive decline remains unclear, anyway women have a 2-fold higher lifetime risk of developing dementia as compared to men [11]. Clinical studies indicate that, estrogen reduction during both menopausal transition and postmenopause can adversely affect brain functions, and in particular brain functions related to verbal memory and verbal fluency [12]. Therefore, in this population already at risk for dementia, a strategy for preventing and delaying cognitive decline is to focus on both the metabolic phenotype [1] but also on the identification of early biomarkers such as peripheral indicators for cognitive decline risk. Moreover, adiponectin that is a plasma protein secreted by adipose tissue [8], and associated to anti-diabetic, anti-inflammatory [13–14], and anti-atherogenic properties [15–16], may result as a biomarker of cognitive decline stage, and its clinical progression [17–18]. During postmenopausal period, in obese and insulin-resistant individuals, serum adiponectin levels are significantly reduced [19–20]. Actually, it is known that in postmenopausal women low plasma levels of adiponectin may be associated with increased prevalence of metabolic syndrome, osteoporosis, and obesity [21], but no studies have evaluated, during this period, the likely relationship about serum adiponectin levels and cognitive performance. Therefore, in this study we aimed to evaluate the relationship between serum adiponectin levels, and cognitive performances in menopausal women, and whether serum adiponectin levels may be differently associated with cognitive performances in normal weight subjects as compared to overweight and obese subjects.

## Materials and methods

### Study population

In this study we screened a study population of 224 postmenopausal women aged 50–66 years. The clinical diagnosis of menopause status was the study eligibility criteria. The diagnosis of menopause was based on three criteria, as recommended by authors of cited paper [22]: self reported menstrual characteristics (last menstruation > 1 year ago) confirmed by blood follicular stimulating hormone (FSH) level (˃20 IU/L) and estradiol level (≤30 pg/ml).

Thirty-six women met exclusion criteria. Exclusion criteria included surgical menopause, hormone replacement therapy or oral contraceptives, hypertension, diabetes mellitus and metabolic syndrome, anemia and/or pulmonary disease and/or cancer, or recent acute illness, severe cognitive decline and/or Alzheimer dementia, or depression history, drugs or alcohol abuse or dependence in the last two years. Finally, among screened population we enrolled 188 postmenopausal women who received evaluation of anthropometric, glycemic, inflammatory and hormonal parameters and performed the psychometric test at our outpatient Geriatric Centre.

According to World Health Organization criteria for overweight and obesity diagnosis [23], obesity was defined as BMI ≥ 30.0 Kg/m^2^ and overweight as BMI≥ 25.0 Kg/m^2^. Therefore, postmenopausal women were then divided into two groups: obese/overweight group (n = 96) and normal weight group (n = 92). After a clear explanation of the study, all women provided written informed consent to participate in the study, which was approved by the Ethical Committee of AOU Second University of Naples.

#### Clinical examination

Clinical and physical examination, anthropometric measurements (weight and height, Body Mass Index-BMI, Waist Hip Ratio-WHR), vital signs (Blood pressure, Hearth rate), nutritional status and life style were evaluated.

#### Laboratory measurements

All blood samples for fasting plasma glucose (FPG), Cholesterol, Interleukin-6 (IL-6), Tumor Necrosis Factor-alfa (TNF-a), C-reactive Protein (CRP) and serum adiponectin levels evaluation were obtained in the morning after overnight fasting and were immediately separated by centrifugation at 1000g, aliquoted and stored at −20°C. Plasma glucose levels were determined by enzymatic colorimetric assay using a modified glucose oxidase-peroxidase method (Roche Diagnostics, GmbH, Mamnhein, Germany). FSH and estradiol levels were determined using MEIA kits, Abbott (sensitivity 1 ng/mL for estradiol and 0.5 mIU/mL for FSH). Serum adiponectin levels were determined using the Quantikine® Human Adiponectin assay (R&D Systems). Serum IL-6 and serum TNF-a were determined in duplicate, using ELISA Assay (Orgenium Diagnostic). CRP was determined using automated turbidimetry.

#### Assessment of cognitive functions

Global cognitive function was assessed with Montreal Cognitive Assessment test (MoCA) [24], used to assess different cognitive domains: attention and concentration, executive functions, memory, language, constructive abstraction, calculation and orientation. The time of administration of the test is 10–15 minutes, the maximum possible score is 30 points; a score equal to or greater than 27 is considered normal. Mild Cognitive Impairment (MCI) was diagnosed according to Petersen’s classification: (a) memory complaint, preferably corroborated by an informant; (b) cognitive impairment in ≥ 1 domains (executive function, memory, language, or visuospatial); (c) normal general cognitive function; (d) intact activities of daily living; (e) no diagnosis of dementia [25]. A diagnosis of MCI was recommended if the MoCA test score is a range between 20 and 26 [24].

Activity functions were assessed by Instrumental Activities of Daily Living (IADL) and the Basic Activities of Daily Living (BADL) while depressive symptoms by Geriatric Depression Scale (GDS short version) [26–27].

## Statistical analysis

Statistical analysis was performed with the use of SPSS software (version 19). All data were presented as mean±SD. A value of P<0.05 was considered significant. A priori power analysis was conducted before the study (G*power) which estimated that a sample size of 135 individuals would achieve adequate power to support the current hypotheses. The inter-group comparison of the metabolic and inflammatory parameters was performed using the Student’s t-test for independent variables. Correlation analysis was performed using a Pearson’s correlation coefficient. Multivariate analysis was used to explore the influence of different variables on MoCA test values including age, BMI, FPG, cholesterol, FSH, Estradiol, TNFa, CRP, IL6 and serum adiponectin levels.

## Results

Anthropometric, metabolic, inflammatory and hormonal characteristics of the study population are reported in Table 1. In the whole group, all participants were overweight and obese (BMI = 27.1± 3.4 kg/m^2^), with appropriate metabolic control, and with mildly higher inflammatory serum levels (Table 1). There was no statistical significant difference about age, systolic and diastolic blood pressure, FPG, and cholesterol serum levels, comparing obese/overweight group to normal weight group. Conversely, obese/overweight group showed lower serum levels of adiponectin (7.5±2.7 v/s 11.2±2.5 microg/ml, p < 0.001) and higher levels of the inflammatory parameters as compared to normal weight group (TNF-a = 1.9±0.3 v/s 1.7±0.2 pg/ml, p < 0.001; CRP = 2.9±0.7 v/s 2.6±0.6 mg/ml, p 0.008; IL6 = 2.2±0.4 v/s 2.1±0.4 pg/ml, p 0.05) (Tab1).

**Table 1:** Anthropometric, metabolic, inflammatory and hormonal parameters of all participants and according to BMI.

	All patients			Obese and overweight group			p	Normal weight group		
	N = 188			N = 96				N = 92		
Age (years)	60.6	±	3.6	60.2	±	4.1	ns	61.1	±	3.1
BMI (Kg/m^2^)	27.1	±	3.4	**30.5**	**±**	**1.2**	**0.001**	**23.6**	**±**	**0.9**
WHR	0.86	±	0.02	**0.88**	**±**	**0.01**	**0.001**	**0.84**	**±**	**0.02**
Systolic blood pressure (mmHg)	118	±	5	117	±	5	ns	118	±	5
Diastolic blood pressure (mmHg)	80	±	3	80	±	3	ns	81	±	4
FPG (mg/dl)	95	±	9	96	±	9	ns	94	±	8
Cholesterol (mg/dl)	175	±	12	176	±	11	ns	173	±	12
TNF-a (pg/ml)	1.8	±	0.3	**1.9**	**±**	**0.3**	**0.001**	**1.7**	**±**	**0.2**
CRP (mg/ml)	2.7	±	0.6	**2.9**	**±**	**0.7**	**0.008**	**2.6**	**±**	**0.6**
IL6 (pg/ml)	2.1	±	0.4	2.2	±	0.4	0.05	2.1	±	0.4
Estradiol (ng/ml)	14.1	±	2.5	13.8	±	2.3	ns	14.2	±	2.7
FSH (ng/ml)	124	±	16	126	±	16	ns	124	±	15
Adiponectin (microg/ml)	9.3	±	3.2	**7.5**	**±**	**2.7**	**0.001**	**11.2**	**±**	**2.5**

In the whole group, cognitive scores revealed a MCI (MoCA test = 25.3±1.7). A significant decrease in global cognitive function occurred (24.3±1.7 v/s 26.3±0.8, p <0.001) when obese/overweight group is compared to normal weight group. Focusing on single cognitive domains, a significant decrease in executive/visuospatial (3.6±0.7 v/s 4.3±0.7, p value <0.001), and in attention functions (4.1±1.1 v/s 4.9±0.7, p value <0.001), occurred in obese and overweight group as compared to normal weight group (Table 2). All participants were well functioning (BADL = 5.7 ± 0.2 and IADL = 6.7 ± 0.9) and were not depressed (GDS score = 3.6 ± 2.4) (data no shown).

**Table 2:** MOCA test values of all participants and according to BMI.

		All patients			Obese and overweight group				Normal weight group		
*Cognitive domains*	*Cut-off*	N = 188			N = 96			P	N = 92		
**Global cognitive function**	***30/30***	**25.3**	**±**	**1.7**	**24.3**	**±**	**1.7**	**0.001**	**26.3**	**±**	**0.8**
Executive/visuospatial functions	*5/30*	**3.9**	±	**0.8**	**3.6**	±	**0.7**	**0.001**	**4.3**	±	**0.7**
Concentration (*naming*)	*3/30*	2.7	±	4.7	2.7	±	0.5	ns	2.8	±	0.4
Attention	*6/30*	**4.5**	±	**0.9**	**4.1**	±	**1.1**	**0.001**	**4.9**	±	**0.7**
Language	*3/30*	2.6	±	0.5	2.5	±	0.5	ns	2.7	±	0.4
Constructive abstraction	*2/30*	1.8	±	0.4	1.7	±	0.4	ns	1.8	±	0.3
Memory	*5/30*	4.1	±	0.7	3.9	±	0.7	ns	4.1	±	0.6
Orientation	*6/30*	5.6	±	0.5	5.5	±	0.5	ns	5.6	±	0.4

As expected, BMI and WHR negatively and significantly correlated with serum adiponectin levels (r = -0.541, p<0.001; r = -0.474, p<0.001). In addition, we found a significant negative relationship between BMI, WHR, TNFa, and a significant positive relationship between serum adiponectin levels and MoCA Global cognitive function (Table 3). Furthermore, only the MoCA executive and MoCA attention functions correlated significantly and negatively with BMI, WHR and significantly and positively with serum adiponectin levels (Table 3). No correlation was found between FSH and estradiol levels and Cognitive functions (Table 3).

**Table 3:** Correlation between anthropometric, inflammatory, hormonal parameters and MoCA test values in all patients.

	MoCA	MoCA	MoCA	MoCA	MoCA	MoCA	MoCA	MoCA
	Global cognitive function	Executive/ visuospatial functio	Concentration (Naming)	Attention	Language	Constructive abstraction	Memory	Orientation
**Characteristics**								
**BMI**	- 0.541**	-0.405**	**- 0.117**	- 0.370**	- 0.157*	**- 0.025**	**- 0.106**	**- 0.081**
**WHR**	- 0.480**	-0.356**	**- 0.081**	-0.273**	- 0.176*	**- 0.067**	**- 0.119**	**- 0.027**
**TNFa**	- 0.247**	**-0.142**	**- 0.078**	**-0.134**	**-0.044**	**- 0.086**	**- 0.113**	**- 0.010**
**CRP**	**- 0.057**	**-0.022**	**- 0.098**	**-0.028**	**-0.011**	**-0.077**	**- 0.029**	**- 0.068**
**IL6**	**-0.135**	**-0.078**	**-0.117**	**- 0.011**	**-0.012**	**-0.082**	**-0.096**	**- 0.035**
**FSH**	**0.769**	**0.582**	**0.894**	**0.395**	**0.927**	**0.134**	**0.894**	**0.441**
**Estradiol**	**0.147**	**0.495**	**0.464**	**0.241**	**0.690**	**0.443**	**0.536**	**0.179**
**Adiponectin**	0.492**	0.209**	**0.173**	0.390**	**0.091**	**0.026**	**0.124**	**0.106**

*p< 0.05

**p < 0.001.

The independent associations of MoCA values with anthropometric, metabolic, inflammatory and hormonal parameters were tested in multivariate analyses (Tables 4 and 5). A first model including age, BMI, WHR, FPG, cholesterol, FSH, estradiol, TNFa, CRP, IL6 levels, as independent variables, explained 39%, 19% and 16% of MoCA Global cognitive function, executive/visuospatial and in attention functions respectively. In such analyses only age and BMI were independently associated with MoCA Global cognitive function, executive/visuospatial and attention functions (Table 4 –Model 1). Adding to this model adiponectin as independent variable, it explained 42%, 19% and 21% of MoCA about Global cognitive function, executive/visuospatial and in attention functions respectively. Therefore, age, BMI and serum adiponectin levels were independently associated with MoCA Global cognitive function, but only serum adiponectin levels were independently associated with MoCA attention (Table 5 –Model 2).

**Table 4:** MODEL 1-Linear multivariate analyses with MoCA total score, MoCA executive score and MoCA attention score as dependent variable.

	*MoCA-Global Cognitive Function*					*MoCA-Executive/visuospatial functions*					*MoCA-Attention*				
	B	SEM	Beta	t	p^value^	B	SEM	Beta	t	p^value^	B	SEM	Beta	t	p^value^
**Age**	**-,088**	**,028**	**-,191**	**-3,161**	**,002**	,005	,015	,023	,329	,743	-,006	,019	-,021	-,302	,763
**BMI**	**-,228**	**,050**	**-,495**	**-4,576**	**,000**	**-,078**	**,027**	**-,356**	**-2,855**	**,005**	**-,121**	**,034**	**-,453**	**-3,564**	**,000**
WHR	-4,662	7,040	-,069	-,662	,509	-1,665	3,851	-,052	-,432	,666	3,332	4,815	,085	,692	,490
FPG	-,012	,011	-,068	-1,126	,262	-,005	,006	-,062	-,878	,381	-,007	,007	-,065	-,910	,364
Cholesterol	-,011	,009	-,077	-1,279	,203	-,001	,005	-,018	-,253	,801	,003	,006	,038	,540	,590
FSH	,009	,006	,087	1,461	,146	,004	,003	,077	1,115	,266	-,001	,004	-,021	-,298	,766
Estradiol	,067	,040	,102	1,677	,095	,017	,022	,054	,769	,443	,033	,027	,086	1,207	,229
TNF-a	-,287	,372	-,052	-,771	,442	-,071	,204	-,028	-,351	,726	,066	,255	,021	,260	,795
CRP	,189	,163	,075	1,155	,250	,133	,089	,111	1,485	,139	,017	,112	,011	,150	,881
IL-6	-,300	,278	-,072	-1,079	,282	-,101	,152	-,051	-,663	,508	,166	,190	,069	,870	,385

**Table 5:** MODEL 2—Linear multivariate analyses with MoCA total score, MoCA executive score and MoCA attention score as dependent variable.

	*MoCA-Global Cognitive Function*					*MoCA-Executive/visuospatial functions*					*MoCA-Attention*				
	B	SEM	Beta	t	p^value^	B	SEM	Beta	t	p^value^	B	SEM	Beta	t	p^value^
**Age**	**-,072**	**,028**	**-,156**	**-2,585**	**,011**	,004	,016	,018	,250	,803	,007	,019	,025	,360	,719
**BMI**	**-,192**	**,050**	**-,416**	**-3,794**	**,000**	**-,080**	**,028**	**-,368**	**-2,845**	**,005**	-,053	,014	-,1346	-1,706	,057
WHR	-2,827	6,934	-,042	-,408	,684	-1,793	3,877	-,056	-,462	,644	4,779	4,707	,121	1,015	,311
FPG	-,008	,011	-,043	-,712	,477	-,006	,006	-,065	-,919	,359	-,003	,007	-,030	-,433	,665
Cholesterol	-,011	,008	-,074	-1,259	,210	-,001	,005	-,018	-,258	,797	,003	,006	,042	,607	,545
FSH	,010	,006	,096	1,637	,103	,004	,003	,076	1,092	,276	-,001	,004	-,009	-,133	,895
Estradiol	,054	,040	,082	1,374	,171	,018	,022	,057	,802	,423	,023	,027	,060	,853	,395
TNF-a	-,156	,368	-,028	-,423	,673	-,081	,206	-,031	-,392	,696	,170	,250	,053	,680	,497
CRP	,178	,160	,071	1,111	,268	,133	,090	,112	1,489	,138	,008	,109	,006	,078	,938
IL-6	-,275	,273	-,066	-1,007	,315	-,103	,153	-,052	-,673	,502	,186	,185	,077	1,001	,318
**Adiponectin**	**,103**	**,038**	**,205**	**2,838**	**,005**	-,007	,021	-,030	-,353	,724	**,084**	**,025**	**,278**	**3,297**	**,001**

## Discussion

The major finding of this study is that a significant positive association may exist between serum adiponectin levels and better cognitive function in postmenopausal women. We showed that cognitive domain of attention function is significantly, and positively associated with serum adiponectin levels, and independently associated with obesity. These findings underscore the relevance of obesity, and overweight in worsening of cognitive function in menopausal women. On the contrary, an unexpected relevant result was the association between serum adiponectin levels, and only the best performances at attention function.

In fact, in most studies obesity and overweight in postmenopause women, are associated with increased risk of cognitive impairment, and dementia [1–7]. Surprisingly, although WHR should be a more sensitive indicator of obesity in comparison with BMI, because the WHR method assesses fat distribution, we showed a significant correlation for WHR and BMI with global cognitive function(Table 3) and a significant correlation for BMI, but not for WHR, with global cognitive function (Tables 4 and 5). Probably our results depend on a smaller and normal range waist values (mean value of waist = 81 cm) and bigger hips values (mean value of hips = 94) of the enrolled women. Scientists believe it is better to have a smaller waist and bigger hips than having all the weight around the middle area. In fact, central obesity defined by WHR is a higher risk factor than BMI-defined obesity, particularly in the presence of central fat distribution [28].

However, the mechanisms underlying the linkage between menopause, obesity and cognitive function are not well understood, and likely involve multiple pathways interacting one with each other [8]. It is well known that in postmenopause women the direct or indirect effects of obesity on cognition are protective by increased endogenous estrogen levels [9–10,14]. In contrast, other studies have emphasized that metabolic conditions observed in obesity, such as oxidative stress alterations, mitochondrial dysfunction, and hyper-inflammation, may be potential mediators in the development and progression of cognitive decline. These effects may contribute in turn to progression of white matter disease, independently by higher and/or lower levels of estrogen [29]. In particular, a possible mechanism for cognitive decline in postmenopausal women may be brain inflammation due to the response of the immune system to decreased levels of estrogen [29–30].

In our study estrogen serum levels were not related to cognitive performance while inflammation and obesity/overweight status were associated significantly and negatively associated to lower levels of the scores obtained from the measure of global cognitive status (MoCA test). Theseresults may mean that, the estrogens effect are not specific on cognitive functions, and/or that the association with cognitive functions may be only spurious. Conversely, our findings are consistent with the hypothesis that low-grade systemic inflammation tone, and obesity may be associated with MCI. Obesity, in turn, accompanied by a proinflammatory condition, stimulate the production of cytokines and of adipocyte-derived hormonal substances. These events can be the key to explaining the existence of a relationship between obesity and cognitive decline [13–14]. Among the various molecules secreted by adipose tissue, adiponectin is known for a long time. Among its many functions, adiponectin is involved in the modulation of glucose metabolism and inflammation [13–14,31] and, at the cerebral level, adiponectin plays a significant role in the cognitive functions disorders [18]. Although in menopause its serum levels may be reduced in obese women and in women affected by insulin resistance and metabolic syndrome [19–21], there are actually no studies evaluating in menopausal women the possible links of serum adiponectin levels and cognitive performance.

Our findings suggest that lower levels of adiponectin may be associated with cognitive dysfunction, independently by obesity. We theorized that, serum adiponectin levels, independently of obesity, could indirectly influence cognitive performances through modulation of several interrelated systemic factors. Indeed, adiponectin receptors are widely distributed in the central nervous system [32–34]. A relevant message of the study is the strong correlation between higher serum levels of adiponectin, and cognitive domains, such as attention, and executive functions. This suggest that adiponectin could preferentially affect these domains and/or the traditional relationships between adipose tissue, adipocytokines and inflammatory biomarkers in the setting of cognitive decline. Although the changes in attentional process and in executive function are not as great as the changes in everyday memory, they can affect all performances [35–36]. Executive function refers to integrative functions of higher cognitive processes that can modulate both cognitive and behavioral components. This modulative effect is necessary for the control of attentional resources, which are at the basis of the ability to manage independent activities of daily living [35–36]. Attentional process may be considered an anatomical network that influences the operation of other brain networks. Attentional process has the function to carry out more than one task at the same time [35–36]. Although the specific definitions of attention and executive function are complex, it may be interesting to know how an aging affects these mechanisms. Indeed, usually a global cognitive evaluation is not performed in clinical practice, because it takes many hours and/or it is performed using the Mini-Mental State Examination (MMSE) [24], which is inadequate to identify a MCI, due to poor sensitivity, lack of executive functions evaluation items, and limited performance range performed usually by normal individuals. It is weel known that MMSE is more specific for patients already suffering from dementia [24]. Conversely, it would be advantageous to identify potential early markers, or peripheral indicators, and/or biomarkers able to predict early risk of cognitive decline [24]. We decided to use the MoCA test for the assessment of cognitive status [24]. Although MMSE also explore cognitive functions, we used the MoCA test as the best battery test to assess a greater number of cognitive domains. The MoCA test is therefore more challenging test, including the assessment of executive functions, attentional capacity, complex visual-spatial functions and complex type speech, being able to allow the identification also only of a mild impairment of these features [24]. Using this test, we found that the major determinant of attentional capacity was just serum adiponectin level. The data shown are of great interest as they indicate that adiponectin could represent an initial marker of cognitive decline. Unfortunately, there are no clinical studies about how adiponectin affects the global cognitive state, attention and executive functions. In clinical practice, it is helpful to emphasize to women that scientific evidence suggests us, that cognitive and attention declines are possible, and very frequent in menopause status. For this reason we may speculate, that this process has to be prevented by the dosage of possible early biomarkers of cognitive decline. Lastly, since adiponectin represents a therapeutic target for some pathologies [37], the activation of its receptors could be a promising way also of cognitive decline modulation. Finally, the present study has several limitations that must be considered. First, this is a cross sectional study showing only an association between serum adiponectin levels, and better cognitive parameters, preventing to affirm a cause effect relationship. Second, this study includes a small sample size of the participants. However, the findings need to be confirmed in larger and longitudinal study.
